# The cumulative effect of various social activities and depression: a longitudinal study in Chinese older adults

**DOI:** 10.3389/fpubh.2025.1570653

**Published:** 2025-06-18

**Authors:** Sixiang Tao, Ming Huo, Hongzhao Wang

**Affiliations:** ^1^Faculty of Physical Education, Huainan Normal University, Huainan, China; ^2^Faculty of Education, National University of Malaysia, Bangi, Malaysia; ^3^Faculty of Rehabilitation Engineering, University of Health and Rehabilitation Sciences, Qingdao, China

**Keywords:** social activity, participation, depression, older adults in China, longitudinal study

## Abstract

**Objective:**

While depression is linked to social activities, few studies comprehensively examine the cumulative and diverse effects of social activity trajectories. Study investigates the long-term relationship between the progression of depression and the patterns of changes in social activity participation.

**Method:**

This study adopted a follow-up research design and analyzed data from three periods of the China Health and Aged Care Tracking Survey (CHRLS) between 2015 and 2020. Data from 3,762 participants aged 60 and older who participated in CHARLS between 2015 and 2020 were included in the analysis. Depressive symptoms were measured by using the Epidemiological Studies Depression Scale (CES-D). A Kaplan–Meier survival analysis was performed, followed by a Cox regression analysis, to identify the interactions between depression and the trajectory of patterns of changes in social activity participation.

**Results:**

In the baseline survey of the third wave study (2015), the prevalence of depression was 24.9%. This increased significantly at each subsequent follow-up, reaching 28.7% at the fourth wave and 32.7% at the fifth wave. Survival analyses showed a statistically significant protective effect of Internet use and leisure-oriented social activities on the prevention of depression after adjusting for confounders such as demographic characteristics, health status, and health behaviors.

**Conclusion:**

Continued participation in recreational social activities and use of the Internet are strongly associated with effective resistance to depression.

## Introduction

As the aging trend intensifies, the policy framework of “Active Aging” has emerged. The “Active Aging” policy framework proposed by the WHO comprises three basic pillars, which offer three primary directions for active aging initiatives: health, participation, and security (1). This framework system focuses on both the physical and mental health and quality of life of older persons and highlights the critical role of social participation for the older population. Actively participating in social activities, giving play to the value of the older adult, and realizing social integration are important manifestations of active aging. According to behavior or activity theory, when the older adults are separated from social groups and familiar work environments, encouraging their participation in social development within the bounds of their abilities can alleviate depression resulting from disconnection from societal progress and the disruption of their social roles (Human behavior and thinking are the result of interaction with the external environment, that is, the formation of stimulus–response connection. In this study, social activity is the stimulus and depression are the response) (2). Research data show that more than 95 million people have depression in China, and depression is a common but often neglected phenomenon among the older adult (3).

Depression has a significant impact on the health of older people, increasing their risk of cardiovascular disease, diabetes and cognitive impairment (4). Additionally, it may contribute to sleep disturbances, changes in appetite, and weight fluctuations. Depression, as a major public health problem, has far-reaching implications at both the individual and societal levels. It has been identified as a leading cause of disability globally and imposes a considerable burden on healthcare systems (5). Some studies have highlighted the global prevalence, burden, and treatment efficacy of depression, emphasizing its critical role as a public health issue (6, 7). Scholars have found that social activity participation plays a positive role in alleviating depressive symptoms, while depression also affects the level of social activity participation in the older adult (8, 9).

Social participation is an important channel for achieving active aging. Studies indicate that it is beneficial for promoting the physical health of the older adult and can effectively reduce their levels of depression (10).

**Figure 1:**
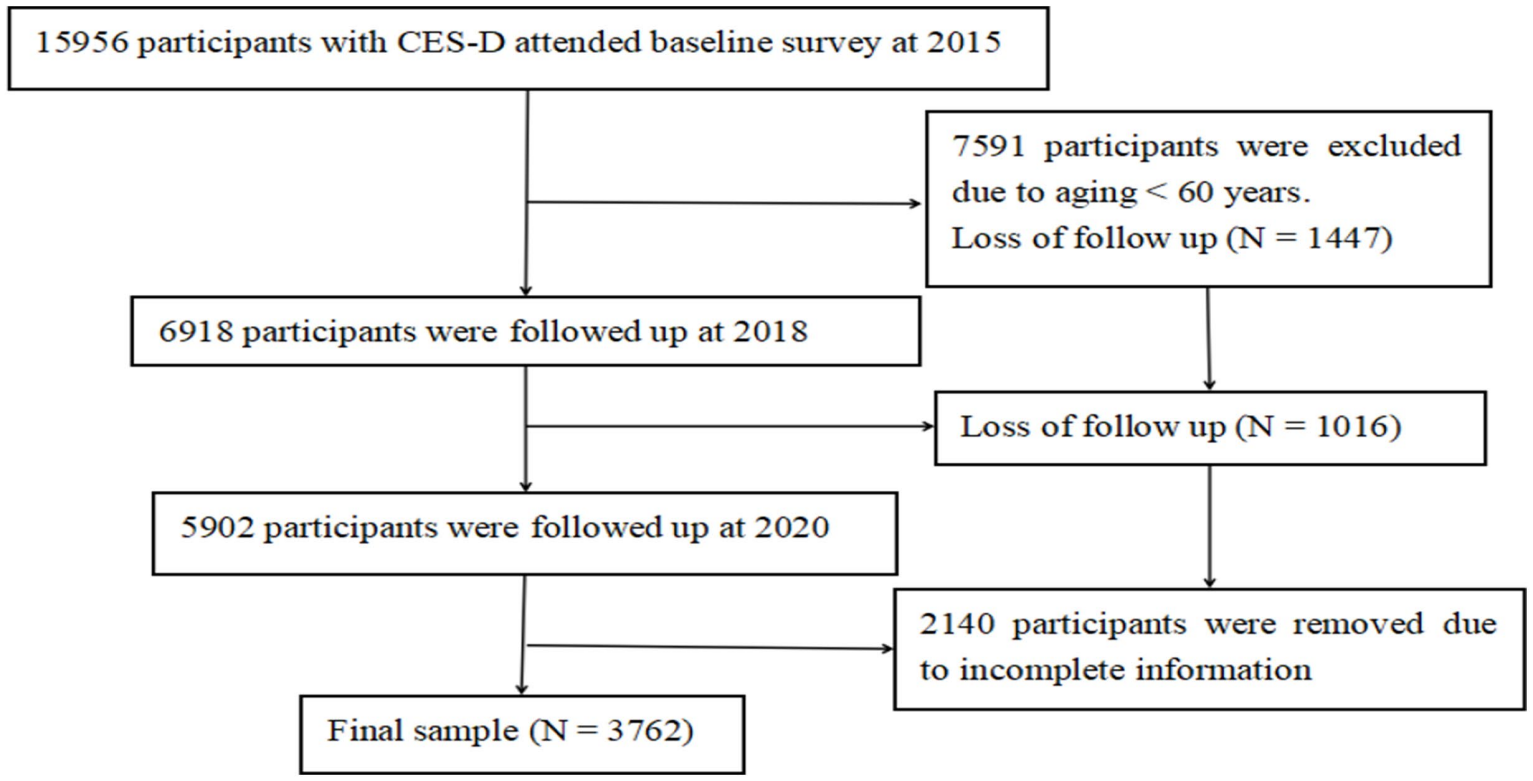
Flowchart of participant.

Some authors have noted that social participation can be categorized into various forms, including formal, informal, productive, consumptive, interpersonal, virtual, face-to-face, and community/social/citizen participation, as well as community involvement and personal relationships. However, the most effective type of social participation for combating depression in older adults is not well documented (11, 12). Consequently, it is significant to recognize that different forms of social engagement can have varying impacts on health (13, 14). Furthermore, the relationship between social participation and health is influenced by factors such as socioeconomic status, social support, and individual health conditions (15). Building upon previous research findings, it is necessary to hypothesize that the impact of social activities on depression may vary. Given the multifaceted nature of social activities, evaluating a single indicator or conducting cross-sectional analysis is insufficient to fully capture the characteristics of population social activity. Such approaches also overlook the comprehensive coexistence and synergistic effects of social activities on the development of mental illness. Particularly for the outcome state of complex traits, further research is essential to fully understand the mechanisms by which social status influences depression. Additionally, there is a need to develop effective interventions aimed at reducing disparities in mental health outcomes associated with social activity status. In accordance with this, the present longitudinal study endeavors to examine the association between trajectories of change in social activity status and the evolution of depressive symptoms. It proposed that changes in the trajectory of social activity states may be related to the emergence or aggravation of depressive symptoms (Figure 1).

## Subjects and methods

### Study population

This investigation encompassed 3,762 older adult participants aged 60 and over, sourced from the CHARLS data survey ranging from 2015 (Wave 3) to 2020 (Wave 5).

Ethical considerations The CHARLS survey has been approved by the Biomedical Ethics Committee of Peking University for each cycle. The fieldwork plan for this phase of the household questionnaire survey has been approved, with the approval number: IRB00001052-11015. Throughout the field survey process, each individual who agrees to participate in the study must sign two copies of the informed consent form. One copy is retained by the participant, and the other is archived at the CHARLS office and filed in PDF scanned form. This study has followed the Strengthening the Reporting of Observational Studies in Epidemiology (STROBE) guidelines.

### Data collection

Demographic variables were collected, including age, gender, marital, residence, and income at baseline.

The Epidemiological Studies Depression Scale (CES-D) was used to assess depression, which has been used to measure depressive symptoms in the older adult (16). The following 10 questions are about how you felt and behaved last week. Each question has the same set of answers, including rarely or never, not too much, sometimes, or half of the time, most of the time. Please choose the appropriate answer. The top 10 questions are listed: perplex, impaired concentration, dejection, exhaustion, expectation, fear, poor sleep, happiness, loneliness, and stagnation. The 7th and 8th items are reverse scoring. The scale options consisted of 4 levels and were assigned: “rarely or none of the time = 0,” “some or few times = 1,” “occasionally or moderate number of times = 2,” “most or all of the time = 3”; The total score ranges from 0 to 30, with scores ≥ 12 were defined as having depressive symptoms (16, 17).

The evaluation of social activities (SAs) includes the following aspects: SA 1. interaction with friends; SA 2. participation in mahjong, chess, card games, or going to community clubs; SA 3. providing assistance for those who do not live together or are not paid; SA 4. participation in sports, social or other types of clubs; SA 5. participating in activities of community-related organizations; SA 6. volunteering, charity work, or caring for patients or disabled people who are not paid; SA 7. participation in education or training courses; SA 8. Internet activities: chatting, reading news, watching videos, playing games, fund management, mobile payment; SA 9. others. Questions regarding participation in social activities include: “Have you participated in the following social activities in the past month?” and “How often did you do the activities mentioned above over the past month?.” Participants who answered “yes” to the question were defined as having some kind of social activity.

Assessment of health status began with self-report, covering respondents’ self-assessment of their overall health, and questions about a range of pain, falls, physical disability, and chronic disease diagnoses raised by physicians. In addition, assessments of activities of daily living (ADLs), and instrumental activities of daily living (IADLs). Questions about physical dysfunction included “Do you have difficulties with activities such as dressing, bathing, eating, getting up, using the toilet, doing housework, cooking, shopping, making phone calls, taking medication, and managing finances due to health and memory reasons? (db001: Difficulty with Dressing; db003: Difficulty with Bathing or Showering; db005: Difficulty with Eating; db007: Difficulty with Getting into or out of Bed; db009: Difficulty with Using the Toilet; db011: Difficulty with Controlling Urination and Defecation; db012: Difficulty with Household Chores; db014: Difficulty with Preparing Hot Meals;db016: Difficulty with Shopping for Groceries; db018: Difficulty with Making Phone Calls; db020: Difficulty with Taking Medications; and db022: Difficulty with Managing Money.).” Participants who answered “yes” to the question were defined as having some kind of physical disability.

The health-related behaviors in this study include smoking, drinking, and physical exercise. The question about smoking was “Have you ever smoked? (Including cigarettes, pipes, hookahs, or chewing tobacco).” Participants who answered “yes” to the question were defined as having smoking behavior. Questions about drinking were “Did drink alcohol over the past year, including beer, wine, rice wine, yellow wine, white spirit, or medicinal liquor, etc.?” and “How often did they drink?.” Questions about physical exercises were “Is doing physical activity due to work needs, recreational activities, physical exercise, or something else? The respondent can choose one or multiple options from: 1. Work needs; 2. Entertainment; 3. Physical exercise; 4. Other.

### Statistics analysis

Use Stata 18 and SPSS 25 for overall data statistics. Record data using frequency and percentage and use chi-square analysis for data comparison. The *p* value of the comparison between variables is less than 0.05, which is statistically significant. In survival analysis, use the Kaplan–Meier method to analyze the development of data trends during follow-up, and use Cox regression to analyze the cumulative risk ratio. In survival analysis, test data robustness by swapping dependent and independent variables in Cox regression.

## Results

### Characteristics of study participants at baseline

Table 1 presents the socio-demographic characteristics of depression and no-depression groups at the baseline of Wave 3. Slightly more than half of the participants were male (53.4%, *n* = 2010). The prevalence of depression was 24.9%. Depressive participants were not significantly older than non-depression groups (*p* = 0.17). Statistics showed a significant difference in the incidence of depression based on gender, marital, residence, and annual income (all *p*-values were <0.01). The incidence of smoking and drinking is significantly lower in the depression group compared to the non-depression group (all *p*-values were < 0.01), while there is no significant difference between the two groups in terms of physical exercise (*p* = 0.3). In terms of health status, physical functional disability, falls, pain, and chronic disease depression groups are all higher than the non-depression group (all *p* values were less than 0.05). However, cigarette smoking (*p* < 0.01) and alcohol consumption (*p* = 0.00) were significantly lower in the depressed group than in the non-depression group. In social activities, there is no significant difference between the depression group and the non-depression group in items SA 1, SA 3, SA 6, and SA 7 (1. Interacted with Friends; 3. Provided Help to Others Who Do Not Live with You or Did Not Pay You for Help; 6. Done Voluntary, Charity Work or Cared for A Sick or Disabled Adult Not Live with You or Did Not Pay You for Help; 7Attended An Educational or Training Course. All *p*-values were more than 0.05).

**Table 1:** Basic characteristics of participants in the non-depression group and the depression group at Wave 3 (*N* = 3,762).

Total (*N* = 3,762)	Non-depression group	Depression group		X2	*p*
	(*n* = 2,824, 75.1%)	(938, 24.9%)			
Demographic background					
Age	60–75 (3122)	74.6	25.4	1.85	0.17
	≧76 (640)	77.2	22.8		
Gender	Male (2010)	81.4	18.6	93.76	0
	Female (1752)	67.8	32.2		
Marital	Yes (2230)	77.8	22.2	21.9	0
	No (1532)	71.1	28.9		
Residence	City (445)	86.5	13.5	35.85	0
	Town (1516)	74.5	25.5		
	Village (1801)	72.8	27.2		
Income	0 (1496)	72.3	27.7	85.63	0
	1–2000 (1377)	70.6	29.4		
	≧2001 (889)	86.7	13.3		
Health status					
Development disabilities	Yes (1004)	61.5	38.5	135.58	0
	No (2758)	80	20		
Fall	Yes (736)	49.5	50.5	320.64	0
	No (3026)	81.3	18.7		
Pain	Yes (458)	63.1	36.9	39.9	0
	No (3304)	76.7	23.3		
Chronic diseases	Yes (2198)	73.7	26.3	5.61	0.02
	No (1564)	77	23		
Health-related behaviors					
Drinking	Yes (966)	80.1	19.9	17.64	0
	No (2796)	73.3	26.7		
Smoking	Yes (1150)	79.5	20.5	17.22	0
	No (2612)	73.1	26.9		
Physical exercises	Yes (713)	77.3	22.7	2.3	0.3
	No (3049)	74.5	24.5		
Social activities					
1	Yes (1241)	76.2	23.8	1.34	0.25
	No (2521)	74.5	25.5		
2	Yes (783)	82.1	17.9	26.29	0
	No (2979)	73.2	26.8		
3	Yes (523)	76.9	23.1	1.05	0.36
	No (3239)	74.8	25.2		
4	Yes (303)	81.5	18.5	7.33	0.01
	No (3459)	74.5	25.5		
5	Yes (114)	86.0	14.0	7.46	0.01
	No (3648)	74.7	25.3		
6	Yes (135)	77.8	22.2	0.55	0.46
	No (3627)	75.0	25.0		
7	Yes (16)	87.5	12.5	1.33	0.25
	No (3746)	75.0	25.0		
8	Yes (114)	90.4	9.6	14.67	0
	No (3648)	74.6	25.4		
9	Yes (42)	88.1	11.9	3.85	0.06
	No (3720)	74.9	25.1		

1: SA1 Interacted with friends.

2: SA2 Played Ma-Jong, played chess, played cards, or went to community club.

3: SA3 Provided help to others who do not live with you or did not pay you for help.

4: SA4 Went to a sport, social, or other kind of club.

5: SA5 Took part in a community-related organization.

6: SA6 Done voluntary, charity work or cared for a sick or disabled adult not live with you or did not pay you for help.

7: SA7 Attended an educational or training course.

8: SA8 Activities with internet: chatting; reading news; watching videos; playing games; money management; payment with mobile phone.

9: SA9 Others.

Disabilities: basic daily activity limitations and instrumental of daily activity limitations.

In the five-year follow-up after taking Wave 3 as the baseline, there were trends of increasing incidence of probability over time for depression (Wave 3 = 24.9%, Wave 4 = 28.7% and Wave 5 = 32.7%; X2 = 56.05, *p* < 0.01.) and SA8 with internet (Wave 3 = 3.0%, Wave 4 = 5.3 and Wave 5 = 18.4; X2 = 636.83, *p* < 0.01); and there were decreasing incidence of SA 1, SA 2, SA 4, and SA 6 (SA 1. Interacted with Friends: Wave 3 = 33.0, Wave 4 = 31.8, and Wave 5 = 29.5; X2 = 11.33, *p* < 0.01. SA 2. Played Ma-Jong, Played Chess, Played Cards, or Went to Community Club: Wave 3 = 20.8, Wave 4 = 18.3, and Wave 5 = 15.9; X2 = 29.69, *p* < 0.01. SA 4. Went to A Sport, Social, or Other Kind of Club: Wave 3 = 8.1, Wave 4 = 5.8, and Wave 5 = 5.2; X2 = 28.53, *p* < 0.01. SA 6. Done Voluntary, Charity Work or Cared for A Sick or Disabled Adult Not Live with You or Did Not Pay You for Help: Wave 3 = 3.6, Wave 4 = 3.0, and Wave 5 = 1.8; X2 = 24.34, *p* < 0.01).

### Five-year follow-up study on the cumulative effects of depressive symptoms and social activities

Through Kaplan–Meier univariate analysis, three insignificant variables of social activities (1, 3, and 6) were excluded, and then Cox proportional regression analysis was performed (Table 2). In this regression model, the depression variable was taken as the state variable, and the three follow-up visits from Wave 3 to Wave 5 were taken as the time variable. The demographic and sociological indicators, health indicators, and other behavioral indicators were taken as covariates to form this brief model. Social activities of SA 2 (Played Ma-Jong, Played Chess, Played Cards, or Went to Community Club, OR = 0.85, CI: 0.77–0.95, *p* < 0.01), SA 4 (Went to A Sport, Social, or Other Kind of Club, OR = 0.85, CI: 0.72–0.99, *p* < 0.01), SA 5 (Took Part in A Community-Related Organization, OR = 0.71, CI: 0.53–0.95, *p* < 0.01), SA 8 (Activities with Internet: Chatting; Reading News; Watching Videos; Playing Games; Money Management; Payment with Mobile Phone, OR = 0.49, CI: 0.42–0.56, *p* < 0.01), and SA 9 (Others, OR = 0.67, CI: 0.49–0.91, *p* < 0.01) are protective factors against depression, after adjusting for demographic factors in the model 1; Social activities of SA 2 (Played Ma-Jong, Played Chess, Played Cards, or Went to Community Club, OR = 0.86, CI: 0.78–0.95, *p* < 0.01), SA 5 (Took Part in A Community-Related Organization, OR = 0.73, CI: 0.58–0.99), SA 8 (Activities with Internet: Chatting; Reading News; Watching Videos; Playing Games; Money Management; Payment with Mobile Phone, OR = 0.51, CI: 0.44–0.59, *p* < 0.01) and SA 9 (Others, OR = 0.73, CI: 0.58–0.99, *p* < 0.01) are protective factors against depression after adjusting demographic factors and health status in the model 2; and social activities of only SA 2 (Played Ma-Jong, Played Chess, Played Cards, or Went to Community Club, OR = 0.89, CI: 0.80–0.99, *p* < 0.01) and SA 8 (Activities with Internet: Chatting; Reading News; Watching Videos; Playing Games; Money Management; Payment with Mobile Phone, OR = 0.54, CI: 0.46–0.62, *p* < 0.01) are protective factors against depression, after adjusting for demographic factors, health status, and health-related behavior in model 3.

**Table 2:** Cumulative interaction between depressive symptoms and social activity factors (*N* = 3,762).

Social activities	Model 1	95.0% CI for Exp (B)		Model 2	95.0% CI for Exp (B)		Model 3	95.0% CI for Exp (B)	
	Exp (B)	Lower	Upper	Exp (B)	Lower	Upper	Exp (B)	Lower	Upper
2	0.85	0.77	0.95	0.86	0.78	0.95	0.89	0.8	0.99
4	0.85	0.72	0.99	–	–	–	–	–	–
5	0.71	0.53	0.95	0.73	0.55	0.98	–	–	–
8	0.49	0.42	0.56	0.51	0.44	0.59	0.54	0.46	0.62
9	0.67	0.49	0.91	0.73	0.58	0.99	–	–	–

2: SA 2 Played Ma-Jong, played chess, played cards, or went to community club.

4: SA 4 Went to a sport, social, or other kind of club.

5: SA 5 Took part in a community-related organization.

8: SA 8 Activities with internet: chatting; reading news; watching videos; playing games; money management; payment with mobile phone.

9: SA 9 Others.

The model 1 is the result of controlling for demographic sociological factors.

The model 2 is the result of controlling for demographic sociological factors and health status.

The model 3 is the result of controlling for demographic sociological factors, health status and health-related behaviors.

## Discussion

This study scientifically demonstrates the impact of the diversity and trajectories of social participation on depression in older adults based on the CHARLS data and reveals that the risk of depression in older people gradually increases with age, according to the five-year follow-up. Although there is a relatively clear relationship between social activity participation and depression, there is relatively little evidence regarding the connection between trajectories of depression and social activity participation (18). This present study further demonstrates that ensuring recreational social interaction and adhering to the Internet may be protective against depression or associated with lower depression risk in older people. This result indicates that older adults with recreational social interaction and adhering to the Internet are not more likely to experience long-term high levels of depression. The analysis suggests that in the process of recreational social interaction, the older adult can effectively eliminate feelings of loneliness, depression, and other negative emotions by constructing and maintaining intimate relationships, thereby improving their health status (19). In our study, this might suggest that providing more opportunities for entertainment for the older adult and encouraging them to use the Internet correctly for social activities is a strong guarantee to improve the quality of life of the older adult. Many older adults lack adequate social support e. g. family support, friend interaction, and community services, which may contribute to worsening their depressive symptoms. These results are partially consistent with previous studies on related issues in China and abroad (8, 9, 16).

**Figure 2:**
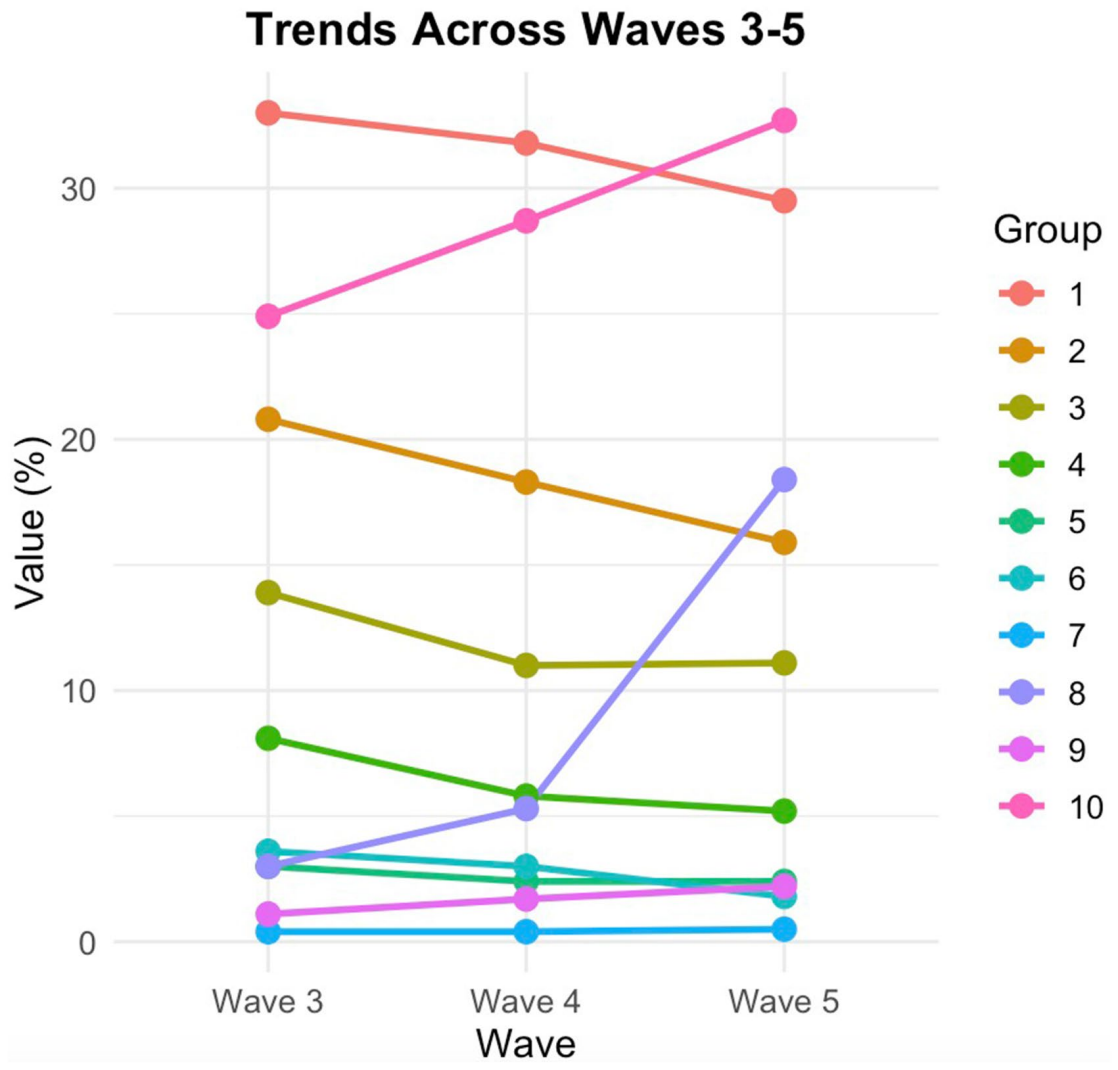
Trajectories in social activities and changes of depression in sequential follow-ups. 1: SA1 Interacted with friends. 2: SA2 Played Ma-Jong, played chess, played cards, or went to community club. 3: SA3 Provided help to others who do not live with you or did not pay you for help. 4: SA4 Went to a sport, social, or other kind of club. 5: SA5 Took part in a community-related organization. 6: SA6 Done voluntary, charity work or cared for a sick or disabled adult not live with you or did not pay you for help. 7: SA7 Attended an educational or training course. 8: SA8 Activities with internet: chatting; reading news; watching videos; playing games; money management; payment with mobile phone. 9: SA9 Others. 10: Depression. 1. X2 = 11.33, *p* < 0.01; 2. X2 = 29.69, *p* < 0.01; 3. X2 = 19.60, *p* < 0.01; 4. X2 = 28.53, *p* < 0.01.;5. X2 = 4.21, *p* = 1.22;6. X2 = 24.34, *p* < 0.01; 7. X2 = 0.50, *p* = 0.78; 8. X2 = 636.83, *p* < 0.01; 9. X2 = 13.57, *p* < 0.01; 10. depression, X2 = 56.05, *p* < 0.01.

Among other findings, two are particularly noteworthy. The firstly, social activity participation is considered to have a positive impact on preventing diseases, especially for the older adult. However, it is necessary to consider the diversity of social activity participation and the direct interactions between various factors. The data from this study show that the activities with internet increased from 3% in 2015 to 18.4% in 2020 over a five-year follow-up period, an increase of more than fivefold (Figure 2). Moreover, the Cox regression analysis indicates that increased activities with internet is the most significant and effective factor in protecting older adults against depression, surpassing leisure and entertainment activities (Table 2). In this study, SA 8, the use of the internet includes: Chatting, Reading news, Watching videos, Playing games, Managing money, Making payments with a smart phone, Using WeChat, and Using WeChat moments, etc. This study did not further divide these contents but agreed to categorize them into one variable as the SA 8 (Tables 1, 2). Innovative construction of age-friendly social media from the perspective of active aging is an important measure to improve the quality of life for the older adult, such as: the service innovation of age-friendly social media, mainly expounding on the functional positioning and service function framework; furthermore, describing the interface innovation of age-friendly social media, mainly the principles of interface innovation and the innovation of interface layout, and so on. The secondly but not the last, the social activity participation of SA 7“Attended An Educational or Training Course” has seen almost no growth over the past 5 years after three follow-ups, and the initial level was already very low (2015: 0.4%; 2018: 0.4%; 2020: 0.5%) (Figure 2). To some extent, this longitudinal data also reflects the serious shortcomings of SA 7 in health education and training for the older adult in the context of active aging. Therefore, the health management department of the older adult should understand the satisfaction of the health education needs of the older adult and related influencing factors, to provide a basis for the formulation of more targeted intervention strategies. Although this study effectively verified the longitudinal association between various factors of social participation and depression, there are still some shortcomings. First, the panel data on social participation and depression in the CHARLS database used in this study contains too much missing data, which may lead to inconsistencies between the results and actual conditions. Second, this study only investigated data from the third round onwards, when individuals entered their older adult stage. Data from those who subsequently entered the older adult stage were not included in this study, inevitably leading to data gaps for these older adults, thus affecting the objectivity of the research results.

Social activity participations are important controllable factors related to depressive symptoms. Previous scholars’ research on the relationship between social activity participation and depression has mostly been based on cross-sectional data, with fewer studies on developmental trajectories (19). This study examines the relationship between longitudinal trajectories of social activity participation and depressive symptoms, which helps us further understand the interplay between the two and assists professionals in formulating optimized health policies.

## Conclusion

The effects of social activity participation on depression are varied, and older individuals often maintain the benefits of social and recreational activity, as well as active participation in social activities with internet, as effective alternatives to antidepressants. Engagement in recreational social activities and Internet use may serve as protective factors against depression in older adults. In the future, technical training programs or social activities should be supported to promote further randomized controlled trial (RCT) research and community intervention experimental research.

## Data Availability

The original contributions presented in the study are included in the article/supplementary material, further inquiries can be directed to the corresponding author.
